# P04.46. Cupping therapy for facial paralysis: a systematic review of randomized controlled trials

**DOI:** 10.1186/1472-6882-12-S1-P316

**Published:** 2012-06-12

**Authors:** H Cao, J Liu

**Affiliations:** 1 Beijing University of Chinese Medicine, Beijing, China

## Purpose

Cupping therapy as part of the traditional Chinese medicine (TCM) is widely used in treating facial paralysis in China. This review focuses on randomized controlled trials (RCTs) to evaluate the therapeutic effect and safety of cupping therapy for facial paralysis.

## Methods

We included all RCTs on cupping therapy for facial paralysis, combination of cupping therapy and other TCM treatments versus other non-TCM therapies were excluded. We searched four databases. All searches ended in December 2010. Methodological qualities of the included trials were evaluated according to criteria of Cochrane Reviewer’s Handbook. Outcome data were summarized using risk ratios (RR) with 95% confidence intervals (CI) for binary outcomes or mean difference (MD) with 95% CI for continuous outcomes. RevMan 5.0.20 software was used for data analyses. Meta-analysis was used if the trials had a good homogeneity, which were assessed by examining I^2^.

## Results

There were 1574 participants in 17 RCTs with generally low methodological quality identified. Two trials were excluded from the meta-analysis due to the special method of intervention. Among the remaining 15 trials, 8 trials used wet cupping, 6 trials used flash cupping, and 1 trial employed medicinal cupping. Meta-analysis showed that the combination of acupuncture and flashing cupping (RR 1.51, 95% CI 1.29 to 1.76, p<0.00001, 5 trials, fixed model) or wet cupping (RR 1.60, 95% CI 1.33 to 1.93, p<0.0001, 6 trials, fixed model) was significantly better than acupuncture alone for a number of cured patients. A combination of cupping therapy and medications was superior to medications alone on reducing average cured time (MD -6.05, 95% CI -9.83 to -2.27, p=0.002, 2 trials, random model). No serious adverse effect was reported in the trials.

## Conclusion

Overall, this review showed the potential effect of cupping therapy in the treatment of facial paralysis. However, further rigorously designed trials on its potential use in other conditions are warranted in the future.

